# Thoracic lymphadenopathies in diffuse systemic sclerosis: an observational study on 48 patients using computed tomography

**DOI:** 10.1186/s12890-022-01837-y

**Published:** 2022-01-25

**Authors:** Arthur Renaud, Raphael Pautre, Olivier Morla, Aurélie Achille, Cécile Durant, Olivier Espitia, Eric Frampas, Christian Agard

**Affiliations:** 1 Internal Medicine Department, Nantes Université, CHU Nantes, 1 place Alexis Ricordeau, 44 000 Nantes, France; 2 Radiology Department, Nantes Université, CHU Nantes, 1 place Alexis Ricordeau, 44 000 Nantes, France

**Keywords:** Systemic sclerosis, Scleroderma, Thoracic lymphadenopathies, Interstitial lung disease

## Abstract

**Background:**

Thoracic multidetector computed tomography (MDCT) is essential for the detection of interstitial lung disease (ILD) in patients with systemic sclerosis (SSc). Thoracic MDCT assessment can reveal the presence of thoracic lymphadenopathies (LAP) whose signification remains uncertain. The purpose of the study was to describe the characteristics and to assess the significance of thoracic LAP in patients with diffuse SSc.

**Methods:**

We conducted a monocentric observational study on adult patients with diffuse SSc, and collected general patient and first thoracic MDCT characteristics, PET-CT and outcome data. Comparisons were made between patients with and without thoracic LAP.

**Results:**

Forty-eight patients were included. There were 30 patients (62.5%) with an ILD and 23 (48%) with at least one thoracic LAP on the first MDCT assessment. Median number per patient of thoracic LAP was 3 [1–8], with a mean size of 11.7 ± 1.7 mm, mainly located in right para-tracheal area (22.8% of the total number of LAP), right hilar area (20.3%), left hilar area (6.5%), and sub-carinal area (15.2%). PET-CT showed lymph node hypermetabolism in 11/15 patients (73.3%) with mean SUVmax at 4 ± 1.3. There were significantly more males (p = 0.002) and more patients exposed to silica (p = 0.001) in patients with thoracic LAP. ILD was significantly more extended according to Goh score (p = 0.03), and using semi-quantitative score for mixed ground-glass reticulation (p = 0.01) and global abnormalities (p = 0.03) in patients with thoracic LAP and ILD. Thirteen patients (27.1%) died during follow-up without significant difference according to the presence or not of thoracic LAP (p = 0.15). There was also no significant difference concerning immunosuppressive treatment initiation (p = 0.17).

**Conclusions:**

Thoracic LAP are common in diffuse SSc and are generally multiple, not bulky, moderately hypermetabolic, and located at the base of the mediastinum lymph node chains. Their presence correlates with the extent of ILD. In absence of ILD, thoracic LAP presence seems to be often explained by silica exposure.

*Trial Registration*: NA.

## Background

Systemic sclerosis (SSc) is a rare and chronic fibrosing auto-immune disease with a heterogenic phenotype and a variable prognosis [1]. Fibrogenesis in SSc is in part explained by Th1/Th2 lymphocyte balance disorder toward Th2 lymphocytes benefit, producing profibrotic cytokines like IL13 and IL4 [2–4]. Environmental factors like silica exposure, and genetic predisposition could be susceptibility factors for SSc [5–8]. Interstitial lung disease (ILD) represents a frequent manifestation of SSc (40–80%), potentially extensive and life-threatening [9]. This complication occurs more often and more seriously in patients with the diffuse cutaneous form of SSc (dSSc), defined as skin fibrosis extended above the elbows and the knees, or when the chest is involved [10, 11].

Thoracic multidetector computed tomography (MDCT) is essential to confirm and monitor ILD during SSc. It brings crucial information concerning the main pattern, but also extension, severity, and evolution of ILD. All patterns of ILD may be encountered, but non-specific interstitial pneumonia (NSIP) is the more frequent, concerning 70–80% of SSc associated ILD (SSc-ILD), followed by usual interstitial pneumonia (UIP) in 8–10% [12]. Thoracic MDCT helps clinicians to guide the treatment of SSc-ILD, which remains challenging despite the use of immunosuppressants [13, 14] or anti-fibrotic drugs like nintedanib [15].

In some cases of SSc, thoracic MDCT analysis identifies thoracic lymphadenopathies (LAP), which signification remains uncertain. Their presence, especially at the initial diagnosis of dSSc, may lead clinicians to prescribe complementary explorations such as nuclear imaging or biopsy, contributing to patient discomfort, iatrogenic risks, and increasing health expenditure.

The literature is poor on the cause, the significance and the outcome of thoracic LAP in SSc. Previous studies suggested a link between SSc-ILD and thoracic LAP, but they did not bring much follow-up information [16–18]. In this context, identification of new markers for extensive and severe ILD in dcSSc is an important issue, and we hypothesized that thoracic LAP might be correlated to ILD severity or prognosis.

## Methods

### Population

We conducted a monocentric observational study in the University Hospital of Nantes, France, regional referral center for systemic and rare auto-immune diseases. We retrospectively analyzed the files of patients with dSSc seen in our center between 2004 and 2019. Patients had to be over 18, with a diagnosis of SSc according to the *ACR/EULAR 2013* criteria [19], in its diffuse cutaneous form, and with at least one thoracic MDCT available in the follow-up.

This study was done in accordance with the *Declaration of Helsinki*, and the French Data Protection Authority and Legislation concerning retrospective studies.

### Data collection

The collection period trended between April 2018 and June 2019. General patient’s characteristics were collected, including epidemiological, clinical, biological, and pulmonary function tests. We collected the presence of thoracic LAP, their size and localization at the time of the first MDCT available. We looked for the presence of ILD pattern and extension according to the Goh score and different interstitial abnormalities according to a semi-quantitative method. Esophagus dilatation was noticed as well. We also collected positron emission tomography-computed tomodentisometry (PET-CT) characteristics and thoracic lymph nodes histological data when available. When available, we analyzed thoracic MDCT during the follow-up in order to describe the evolution of thoracic LAP and ILD over time. Finally, we collected patients’ outcome such as immunosuppressive treatment initiation and death.

MDCT were performed on two different machines: A Siemens “Sensation16” (70mAs, 120 kV, 1 mm thin, reconstruction « field of view» (FOV) of 300 mm, and 16 barrettes) and a Toshiba “Aquilon Prime80” (180mAs, 120 kV, 1 mm thin, reconstruction FOV of 380 mm, and a collimation of 0.5 mm × 80 barrettes) allowing high resolution analysis of the parenchyma. Images were analyzed by two different radiologists experts in thoracic imaging (RP and OM) on a high-resolution gray-scale monitor (Eiza model, 2048 × 1536 pixel).

Thoracic LAP were defined as lymph nodes with a small axis sized ≥ 10 mm. Their localizations were described according to the chest cartography of the *American Thoracic Society* [20]. Hilar lymph nodes were considered as mediastinal. In patients who underwent a PET-CT, we collected LAP metabolism data with the evaluation of « Standardized Uptake Value» (SUV) for the more hypermetabolic lymph node. Evolution was assessed using the first and the last thoracic MDCT available. Thoracic LAP number was considered stable when it was identical at 1 lymph node near, increased when 2 or more lymph nodes were added and decreased when 2 or more lymph nodes disappeared.

In cases of ILD, the MDCT pattern was evaluated: UIP pattern was defined according to current diagnostic criteria [21], and NSIP when ground glass was the dominant characteristic. All other situations were considered as undetermined patterns. ILD extension was evaluated with two different methods. A detailed semi-quantitative method according to Kanzerooni [22]: ground glass alone, mixed ground glass and reticulation, reticulations alone, honey-combing, and all abnormalities in global were separately analyzed. A score for each feature was attributed for every pulmonary segment: 0 when absent, 1 when the extension was between 1–25%, 2 between 26–50%, 3 between 51–75%, and 4 between 76–100%. Scores were assembled to obtain a global score at lung scale trend from 0 to 20 (5 lobes). Goh score corresponds to a global ILD extension assessment using pulmonary function testing data. It was defined as “extended” when ILD was above 20% of pulmonary parenchymatous volume, and as “limited” when below. When it was undetermined, extension was defined as “limited” when force vital capacity (FVC) ≥ 70% and as “extended” when FVC < 70% [23]. Evolution was evaluated between the first and last thoracic MDCT available for global extension using the semi-quantitative method.

Esophagus dilatation was assessed using a method inspired by Richardson et al. [24]. Esophagus light diameter measurement was performed just below the aortic cross in the great axis where the esophagus is the more dilated. It was defined as a maximum esophagus light diameter $\ge$ 9 mm [25].

### Statistical analysis

Continuous variables were described as means (standard deviation) or medians (interquartile range 25–75%), and categorical variables as proportions (%).

We compared clinical, biological, and imaging characteristics between two groups: with or without thoracic LAP at the time of first thoracic CT-scan assessment. We also compared thoracic LAP’ characteristics between two groups according to the presence of ILD.

Proportion comparisons between groups were assessed using Pearson’s Chi^2^ test with systematic Yale’s correction regarding the low group’s number. When the expected theoretical number in the contingency table was ≤ 5, Fisher’s exact test was used. Means comparison between groups was assessed using Student t-test when values respected a normal distribution and variances were considered as equal. If the distribution wasn’t normal, Mann–Whitney test was used. Survival comparison between groups was assessed using log-rank test. The significance threshold was p < 0.05. Statistical analyses were assessed using Graph Pad Prism software version 6.0.

## Results

### Population

**Fig. 1:**
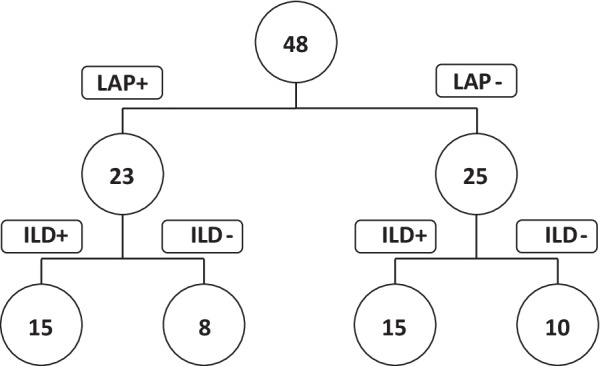
Patients’ distribution among different sub-groups according to initial thoracic MDCT. LAP+: presence of one or more mediastinal lymphadenopathies. LAP−: absence of mediastinal lymphadenopathies. ILD+: presence of interstitial lung disease. ILD−: absence of interstitial lung disease

We included 48 patients, 23 patients (48%) with at least one thoracic LAP and 30 patients (62.5%) with an ILD on first MDCT assessment. The distribution of our patients according to the presence or absence of thoracic LAP and/or ILD on first MDCT assessment is shown in Fig. 1. The general characteristics of the patients are described in Table 1. Majority of patients had anti-topoisomerase 1 antibodies (n = 26), and 15 patients (31%) had anti-nuclear antibodies without any SSc-specific antibodies.

**Table 1:** Comparison between groups according to the presence of thoracic LAP on MDCT initial assessment

Characteristics	Total	LAP+	LAP−	p
	(N = 48)	(N = 23)	(N = 25)	
*Epidemiological*				
Female sex (%)	30 (62.5)	9 (39.1)	21 (84)	0.002
Age in mean* ± SD (years)	52 ± 14	51 ± 10	53 ± 14	NS
Mean disease duration ± SD (years)	2.5 ± 4.1	2.3 ± 4.1	2.8 ± 4.2	NS
Silica exposure (%)	8 (16.7)	8 (34.8)	0 (0)	0.001
Tobacco exposure** (%)	14 (29.2)	8 (34.8)	6 (24)	NS
*Phenotype*				
GERD (%)	47 (97.9)	22 (95.7)	25 (100)	NS
Intestinal involvement (%)	7 (14.6)	4 (17.4)	3 (12)	NS
Ano-rectal involvement (%)	5 (10.4)	1 (4.4)	4 (16)	NS
Joint involvement (fingers/hands/wrists) (%)	21 (43.7)	11 (47.8)	20 (80)	0.03
Muscular involvement (%)	4 (8.3)	1 (4.4)	3 (12)	NS
Raynaud’s phenomenon (%)	46 (95.8)	22 (95.7)	25 (100)	NS
Digital ulcer (%)	26 (54.2)	11 (47.8)	15 (60)	NS
Cutaneous calcinosis (%)	10 (20.8)	3 (13)	7 (28)	NS
Telangiectasia (%)	29 (60.4)	15 (60)	14 (56)	NS
Sicca syndrome (%)	11 (22.9)	6 (-26.1)	5 (20)	NS
Scleroderma renal crisis (%)	9 (18.8)	3 (13)	6 (24)	NS
Pericarditis (%)	5 (10.4)	1 (4.4)	4 (16)	NS
Myocardial involvement (%)	7 (14.6)	6 (26.1)	5 (20)	NS
Pulmonary hypertension (%)	5 (10.4)	3 (13)	2 (8)	NS
Group 1 (PAH) (%)	3 (6.3)	2 (8.9)	1 (4)	NS
Group 3 (%)	2 (4.2)	2 (8.9)	0 (0)	NS
Rodnan score*** ± SD	23 ± 8	22 ± 7.6	24 ± 8.4	NS
ILD (%)	30 (-62.5)	15 (65.2)	15 (60)	NS
*Immunological*				
Anti-Scl70 antibody (%)	26 (54.2)	16 (69.6)	10 (40)	0.078
Anti-centromere antibody (%)	2 (4.2)	1 (4.4)	1 (4)	NS
Anti-RNA polymerase III antibody (%)	4 (8.3)	0 (0)	4 (16)	NS
Anti-U1RNP antibody (%)	3 (6.3)	0 (0)	3 (12)	NS
No antibody specificity (%)	15 (31.3)	6 (26.1)	9 (36)	NS
Mean CRP level in mg/L^♦^ ± SD	18 ± 20	22 ± 19.8	15 ± 20.1	NS
*Pulmonary function test*^♦♦^				
Mean FVC in mL ± SD	3024 ± 985	3185 ± 1025	2981 ± 953	NS
Mean DLCO in % ± SD	65 ± 22	61 ± 21	71 ± 21	NS

LAP = lymphadenopathies; LAP +  = presence of thoracic lymphadenopathies; LAP- = absence of thoracic lymphadenopathies; GERD = gastroesophageal reflux disease; PAH = pulmonary arterial hypertension; ILD = interstitial lung disease; CRP = C-reactive protein; FVC = forced vital capacity; DLCO = diffusion capacity of carbon monoxide

*Age at initial MDCT assessment; **Defined as an active consumption or a smoking cessation with estimated consumption above 10 year-pack; ***Maximal Rodnan score during follow up; ^♦^CRP level at diagnosis; ^♦♦^Pulmonary function test at diagnosis; NS = Non statistically significant

### Thoracic MDCT

Thoracic LAP characteristics are described in Table 2. Seventy-nine thoracic LAP have been counted in the overall initial assessment with, among patients concerned, a median number per patient of 3 (IQR 2–5, maximum 8) and a moderate mean size of 11.7 ± 1.7 mm. Main thoracic lymphatic areas involved were right para-tracheal area (n = 18 LAP, 22.8% of the total number of LAP), right hilar area (n = 16, 20.3%), left hilar area (n = 13, 6.5%), and sub-carinal area (n = 12, 15.2%).

**Table 2:** Thoracic lymphadenopathies on MDCT characteristics at initial assessment

Characteristics	Total
	(N = 23)
Total number of thoracic LAP	79
Per patient in median (IQR) (max–min)	3 (2–5) (1–8)
1 (%)	5 (21.7)
2 (%)	4 (17.4)
3 (%)	3 (13.0)
4 (%)	5 (21.7)
5 (%)	2 (8.7)
6 (%)	2 (8.7)
7 (%)	1 (4.4)
8 (%)	1 (4.4)
Thoracic LAP > 2 (%)	14 (60.9)
Size in mean ± SD (max – min)	11.7 ± 1.7 (10–18)
Localization*	
1R (%)	1 (1.3)
1L (%)	1 (1.3)
2R (%)	3 (3.8)
2L (%)	1 (1.3)
3 (%)	1 (1.3)
4R (%)	18 (22.7)
4L (%)	3 (3.8)
5 (%)	4 (5.0)
6 (%)	4 (5.0)
7 (%)	12 (15.2)
8 (%)	2 (2.5)
9 (%)	0 (0)
10R (%)	16 (20.3)
10L (%)	13 (16.5)
Calcification (%)	6 (12.5)

LAP = lymphadenopathie; CT = computed-tomodensitometry

^*^Localization according to the chest cartography of the American Thoracic Society (20)

ILD characteristics are described in Table 3. The most frequent abnormality found was mixed ground glass-reticulation for 25/30 patients (83%). Pulmonary parenchymal abnormalities other than SSc-ILD were described: one patient had ground glass and nodules with infectious aspect, one patient had features corresponding to pulmonary veno-occlusive disease, one patient presented moderate bilateral pleural effusion and ground glass suggestive of cardiac insufficiency, and one patient known for silicosis had few solid nodules with random distribution. Nodules at the pleuro-scissural interface or located less than 10 mm from the pleura, considered as intra-pleural LAP, were found in 9 patients (19%). No lesions suggestive of neoplasia have been seen in our patients.

**Table 3:** ILD characteristics at initial assessment and comparison according to presence of thoracic lymphadenopathies

Characteristic	Total	ILD with LAP +	ILD with LAP-	p
	(N = 30)	(N = 15)	(N = 15)	
NSIP pattern (%)	20 (66.7)	11 (73.3)	9 (60)	0.7
UIP pattern (%)	4 (13.3)	1 (6.7)	3 (20)	0.6
Undetermined pattern (%)	6 (20)	3 (20)	3 (20)	1
Extended form* (%)	15 (50)	11 (73.3)	4 (26.7)	0.03
Semi-quantitative extension score				
Ground glass alone ± SD	2 ± 3.6	2.4 ± 3.5	1.2 ± 3.6	0.09
Mixed ground glass–reticulation ± SD	5.3 ± 4.1	7.1 ± 3.9	3.8 ± 2.8	0.01
Reticulation alone ± SD	0.6 ± 1.4	0.4 ± 1.1	0.9 ± 1.9	1
Honey combing ± SD	0 ± 0	0 ± 0	0 ± 0	1
Global ± SD	6.6 ± 5.1	8.7 ± 5.1	5.1 ± 3.7	0.03

ILD = interstitial lung disease; CT = computed tomography; NSIP = nonspecific interstitial pneumonia; UIP = usual interstitial pneumonia; LAP +  = presence of thoracic lymphadenopathies on MDCT assessment; LAP- = absence of lymphadenopathies on MDCT assessment; ILD +  = presence of interstitial lung disease on TDM assessment; ILD- = absence of interstitial lung disease on MDCT assessment

*According to Goh score

Concerning the esophagus characteristics, dilatation was present in 38 patients (79.2%) and the mean diameter was 17.1 ± 8.4 mm.

The subset analysis of the 15 patients without any SSc-specific antibodies did not show any specific findings when compared to the 33 others, especially regarding frequence of ILD (73% vs 74%) and percentage of patients LAP + (47% vs 52%).

### Other explorations

PET was performed in 15 patients (31.3%). Indications were the search for underlying neoplasia in 12 patients (80.0%), characterization of thoracic LAP for 2 patients (13.3%), and evaluation before heart transplant for 1 patient (6.7%). Lymph node hypermetabolism was found in 11/15 patients (73.3%), even in the absence of LAP for 4/15 patients (26.7%). SUVmax in overall PET was 4 ± 1.3 with a maximum at 6.

Four mediastinal lymph node biopsies and 1 aspiration cytology have been performed, always in case of neoplasia suspicion. Histological analysis never described granuloma nor tumoral cells. One biopsy revealed primary lymphoid follicles including small-sized lymphocytes with mature chromatin and discrete sinusal histiocytic aspect, one other revealed nonspecific small and medium-sized lymphocytes, and the two last were not contributive because of a lack of lymph node material. The aspiration cytology showed regular, small-sized, mature lymphocytes and anthracosis histiocytes.

### Comparison analysis

General characteristics comparisons between groups according to the presence of thoracic LAP are described in Table 1. There were significantly more males [14 (61.9%) versus 4 (16.0%), p = 0.002], and more cases exposed to silica [8 (34.8%) versus 0 (0.0%), p = 0.001] in patients with thoracic LAP. All patients with thoracic LAP but without ILD were males (n = 8, 100%) and 5 of them (63%) had been exposed to silica, while there were 18 males (37.5%) with 8 (16.7%) exposed to silica in the whole cohort (p < 0.05).

Thoracic LAP characteristics were similar in size (p = 0.74) and number (p = 0.53) between groups according to the presence of ILD.

Comparison of ILD characteristics on MDCT initial assessment, according to the presence of thoracic LAP, are shown in Table 3. ILD was significantly more extended according to the Goh score [11 (73.3%) versus 4 (25.0%), p = 0.03] in patients with thoracic LAP and ILD. Using semi-quantitative extension score, mixed ground-glass-reticulation (2.4 ± 3.5 versus 1.2 ± 3.6, p = 0.01] and global abnormalities [8.7 ± 5.1 versus 5.1 ± 3.7, p = 0.03] were also significantly more extended in patients with thoracic LAP and ILD.

There were no significant differences concerning esophagus characteristics, nor in dilatation proportion [20 patients (87.0%) vs 18 patients (72.0%), p = 0.18], neither in mean diameter (14.8 ± 6.4 vs 13.6 ± 8.2, p = 0.57) between the two groups according to the presence of thoracic LAP.

PET-CT was performed in 9 patients (39.1%) with thoracic LAP and 6 patients (24.0%) without (p = 0.41). Lymph node hypermetabolism was significantly more frequent [8 (88.9%) versus 3 (50.0%), p = 0.05] and SUVmax was almost significantly higher [3.8 ± 1.6 versus 2.2 ± 1.3, p = 0.067] in patients with thoracic LAP.

### Evolution and outcome

Four patients (8.3%) were lost to follow-up. Thirteen patients (27.1%) died during follow up with a mean duration of 6.5 ± 4.8 years: 9 among patients with thoracic LAP and 4 in the absence of thoracic LAP (p = 0.15). No diagnosis of neoplasia or systemic granulomatosis has been made during follow up. Thirty-seven patients (77.1%) had at least one immunosuppressant initiated during the follow up: corticosteroids for 29 (60.4%), mycophenolate mofetil for 25 (52.1%), cyclophosphamide for 18 (37.5%), methotrexate for 12 (25.0%), azathioprine for 2 (4.2%), and rituximab for 2 (4.1%). There was no significant difference concerning immunosuppressive treatment initiation between groups according to the presence of thoracic LAP (p = 0.17).

Overall, 36 patients (75.0%) had at least two thoracic MDCT available in their follow-up, with a mean duration of 4.5 ± 3.6 years, allowing temporal comparison analysis.

**Fig. 2:**
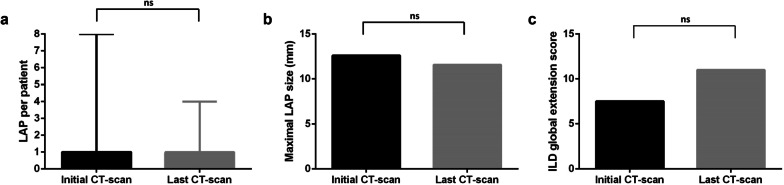
Evolution between first and last thoracic MDCT available in patients with thoracic LAP. **a** Number of thoracic lymphadenopathies per patient in median (with range). **b** Maximum size of thoracic lymphadenopathies in mean (mm). **c** ILD’s global extension in mean according to semi-quantitative method

**Fig. 3:**
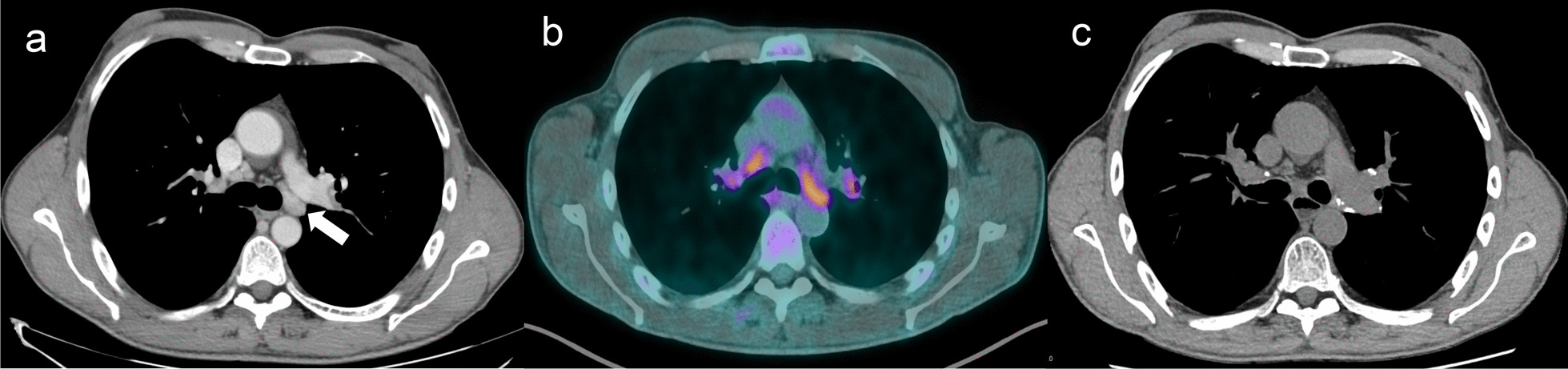
Example of thoracic LAP evolution in one patient exposed to silica. One Thoracic LAP has been revealed in 4L area on first thoracic MDCT assessment (**a**, white arrow) with an hypermetabolism on PET-CT (**b**). At 9 months, a spontaneous regression of the thoracic LAP has been highlighted on thoracic MDCT with occurrence of calcifications (**c**)

Evolution of thoracic LAP characteristics between first and last thoracic MDCT available are shown in Fig. 2. The number of thoracic LAP was stable in 24 patients (66.7%), increased in 8 patients (22.2%) and decreased in 4 patients (11.1%). In one patient exposed to silica with one thoracic LAP in the 4L area found in thoracic MDCT at initial assessment, also hypermetabolic on PET-TDM, the size of the LAP normalized spontaneously in 9 months, associated with occurrence of calcifications (Fig. 3).

Evolution of ILD global extension score between first and last thoracic MDCT available according to the semi-quantitative score among patients with thoracic LAP is shown in Fig. 2.

## Discussion

Our study is the second that provides precise information about thoracic LAP seen on MDCT in patients with dSSc. Almost 50% of our patients had at least one thoracic LAP with a median number of 3 and a moderate mean size of 11.7 ± 1.7 mm. The more frequent thoracic topographies were right para-tracheal (22.8%), right hilar area (20.3%), left hilar area (16.5%), and sub-carinary area (15.2%). Their presence was significantly more frequent in men and in patients exposed to silica, but no significant differences concerning other characteristics have been found between groups, except for joint involvement (arthritis and/or synovitis and/or contracture of fingers and/or hand and/or wrists), more frequently noted in patients without thoracic LAP, whose meaning remains unclear. The number of lymph nodes remained stable or decreased mainly in patients over time. The occurrence of ILD was similar between groups according to the presence of thoracic LAP, and the main pattern was NSIP, as expected. However, ILD was more extended in the presence of thoracic LAP. When PET-CT was available, there was frequently a mediastinal lymph node hypermetabolism (73%) with mean SUVmax measured at 4 ± 1.3 and a trend to be more important in the thoracic LAP group. When available, mediastinal lymph node histological analysis did not show granuloma nor neoplasia. Analysis over time did not show significant evolution of thoracic LAP and differences between groups according to the presence of thoracic LAP concerning ILD.

The prevalence of thoracic LAP in our study was similar to that found in the only study on homogenous population of dSSc patients (58%) [16]. It is also fairly close to the overall prevalence in all forms of SSc, whether diffuse or limited [17]. A discrepancy with the literature was identified regarding the prevalence of thoracic LAP in the presence of ILD, which was only 50% in our study compared to 72% in the study by Weschler et al. and 60% in the study by Bhalla et al. [16, 18]. This last study certainly underestimated the prevalence of thoracic LAP, due to the retained definition of LAP [12 mm minimum minor axis] [18]. Based on this high prevalence, the study by Weschler et al. justified the finding of a higher prevalence of thoracic LAP in the diffuse form than in the limited form of SSc, since this latter is more rarely complicated by ILD [16]. This difference may be explained by a high prevalence of thoracic LAP in patients without ILD in our study (35%) which is higher than other studies (8% and 17%) [16, 17]. This is partly explained by a higher proportion of men in our study (38%), when compared to other cohorts (13–33%), with numerous patients exposed to silica, characteristic which was not taken into account in previous studies. The sole male gender as an epidemiological factor should however be put in perspective: silica is a particle that can be found in certain manual occupations, rather practiced by men, in our study, and all of the 8 patients who had an occupational exposure were men. It is possible that silica exposure may not have been correctly identified in some patients, due to non-professional exposure for example. In a recent study, 16% of patients were significantly exposed to silica, which was assessed by a specific questionnaire on occupational and non-occupational activity. It was associated with the combination of thoracic LAP (OR = 8.09) and was predictive of worsening of pulmonary involvement (OR = 4.7) [26].

In the context of ILD, the literature suggests a mechanism of lymph node hypertrophy reactive to inflammatory activity of the lung, which is supported by pathophysiological postulates and by several correlations established between ILD and thoracic LAP [16, 27]. Our study confirms that the overall extent of ILD is correlated with the presence of thoracic LAP. The study by Weschler et al. also showed a strong correlation between thoracic LAP and the extent of ILD, while Garber et al. also found an increased prevalence of thoracic LAP in each subgroups of patients with increasing ILD extent [16, 17]. These correlations remind what has been shown about thoracic LAP in idiopathic pulmonary fibrosis (IPF), in which the prevalence of thoracic LAP is estimated to be 66–70%, and associated to chronic and advanced disease process [28–30]. Both IPF and SSc-ILD share an inflammatory process responsible for a locoregional environment rich in pro-inflammatory cytokines potentially circulating in the lymphatic network. This lymphatic network, which is highly interconnected and varies from one individual to another, does not allow precise reasoning on a topographical correlation between a segment and a LAP. This diversity is, for example, at the origin of "skip-metastases" in a neoplastic context [31]. However, our study shows that lymph nodes are particularly at the origin of the mediastinum drainage chains, which is consistent with the mechanism of lymph node hypertrophy in question.

Survival was not significantly different in patients with thoracic LAP during follow-up. Moreover, the use of immunosuppressive treatment was similar in groups according to the presence of thoracic LAP. The information on immunosuppressive treatment was important to collect because these treatments may influence ILD, thoracic LAP, and broncho-alveolar cytology. Several studies evaluating broncho-alveolar lavage analysis in SSc patients have shown a tendency to decrease the total number of cells and/or the proportion of granulocytes in patients under immunosuppression [32–34] and in IPF, an impact of immunosuppressive treatment has been described on thoracic LAP [31, 35].

One of our study’s strengths is the homogenous characteristic of our population composed only by patients with dSSc, whether or not associated with ILD. However, this is a retrospective monocentric study on a rare disease, explaining the low number of patients. Furthermore, inaccuracies in the analysis of the extent of ILD may have occurred. The semi-quantitative assessment is by definition an approximation at the discretion of the reader. The boundary between ground-glass and normal parenchyma or the detection of cross-linkages can be subtle and intra/inter-observer agreement has not been studied. Another limitation of the ILD quantification methodology can also be raised: the asymmetry in the number of lung lobes on the right and left is the cause of an overweighting of the right lung score. Some publications have taken this problem into account by, for example, adding a correction factor for each lobe [36]. It should also be mentioned that the first stage gives an identical score (1/4) for very low extent of involvement and for more extensive involvement involving less than 25% of the lobar volume. Finally, silica exposure has not been quantified for its duration or intensity of exposure, which could be clarified in future research so as not to overestimate the secondary nature of the disease. In addition, it would be interesting to compare our data with a control population of patient with exposure to silica but no SSc. And, unfortunately, we do not have information about solvents exposure, another incriminated risk factor for SSc.

## Conclusions

Thoracic LAP are common in dSSc and are generally multiple, not bulky, not extensive nor compressive, moderately hypermetabolic, and located at the base of the mediastinum lymph node chains. Their number are mainly stable in time. Their presence correlates with the extent of ILD. No diagnosis of neoplasia or granulomatosis have been made in the follow-up. In the absence of ILD, thoracic LAP presence seems to be explained by epidemiological factors, such as exposure to silica, frequently involved in secondary dSSc, especially in men.

## Data Availability

The datasets used and/or analyzed during the current study are available from the corresponding author on reasonable request.

## Abbreviations

dSSc: Diffuse systemic sclerosis
ILD: Interstitial lung disease
IPF: Idiopathic pulmonary fibrosis
LAP: Lymphadenopathies
MDCT: Multidetector computed tomography
NSIP: Non-specific interstitial pneumonia
PET-CT: Positron emission tomography-tomodentisometry
SSc: Systemic sclerosis
SSc-ILD: SSc-associated ILD
UIP: Usual interstitial pneumonia
